# Cook County Hospital

**Published:** 1870-01

**Authors:** Curtis T. Fenn


### Hospital Reports.

ARTICLE I.— *Cook County Hospital. Under the care of* CURTIS T. FENN, M.D.

LIGATION OF EXTERNAL ILIAC ARTERY — DEATH. *September* 17.— Dr. Powell tied the left external iliac for diffused traumatic femoral aneurism, in the case of J. L., a stalwart Indiana farmer, aged 28, admitted to a private ward, September 13, 1869.

The patient died on the 20th, with symptoms of peritonitis.

During the last year of the war, he received a gunshot wound in the lower part of the left Scarpa's triangle. The ball glanced upward, and was lost. The wound healed.

In August, 1869, after a hard day's work, cradling in the harvest-field, he perceived a small swelling in the region of the cicatrix. It came in a day, and without pain. The next day, he felt "a rushing" there. The tumor increased with great rapidity from this beginning, and on the day of the operation presented a terrible aspect. A diffuse, ovoid, firm elastic mass occupied the anterior and inner aspect of the upper third of the thigh. Its centre was over the lower portion of Scarpa's triangle.

The enlargement was limited above by the abdomen, extending a little above Poupart's ligament, and diminished downward, reaching two-thirds of the way to the knee. The circumference of the thigh at its largest portion was nineteen and one-fourth inches greater than the corresponding measurement of the opposite side. A pulsating movement of the surface was visible across the amphitheatre. The skin was hot and shining. A rosy tint over the centre was deepening to red. A thrill of great force was communicated, when the hand was laid on the tumor. A

loud rushing sound greeted the ear by auscultation; compression of the femoral artery, above the pubis, caused it to cease. The limb was comfortable when at rest, the circulation good, the temperature about normal, and, aside from the sense of weight and uselessness which it gave, the patient suffered no inconvenience. There was little general loss of flesh or strength, yet mental anxiety and suffering were depicted.

At a council of surgeons, it was decided that ligature of the external iliac afforded the best chance of arresting the disease.

Ether was administered. The patient came under the influence of the anæsthetic with difficulty. His breathing was accompanied by such spasmodic abdominal exertion as to interfere with the operation and endanger the peritoneum as soon as it became exposed. The incision was according to Abernethy's method. The application of the ligature was tedious, owing to imperfect anæsthesia, and the consequent hernial protrusion of the peritoneum, in spite of all efforts to retain it. The wound was dressed with sutures, adhesive strips and cold compress. Difference in the temperature of the extremities was well marked. Artificial heat was applied to the affected side, and opium given to keep the patient quiet, with beef tea for nourishment.

He died the third day, with symptoms of peritonitis.

*Post-mortem* examination revealed the aneurism springing from the trunk of the profunda femoris. The ball supposed to be lodged in the region was not found, nor could its track be traced.

Dr. Powell regards this case one of great interest, and intends to publish it at the proper time.

